# GenBase: A Nucleotide Sequence Database

**DOI:** 10.1093/gpbjnl/qzae047

**Published:** 2024-06-24

**Authors:** Congfan Bu, Xinchang Zheng, Xuetong Zhao, Tianyi Xu, Xue Bai, Yaokai Jia, Meili Chen, Lili Hao, Jingfa Xiao, Zhang Zhang, Wenming Zhao, Bixia Tang, Yiming Bao

**Affiliations:** National Genomics Data Center, Beijing Institute of Genomics, Chinese Academy of Sciences and China National Center for Bioinformation, Beijing 100101, China; CAS Key Laboratory of Genome Sciences and Information, Beijing Institute of Genomics, Chinese Academy of Sciences and China National Center for Bioinformation, Beijing 100101, China; National Genomics Data Center, Beijing Institute of Genomics, Chinese Academy of Sciences and China National Center for Bioinformation, Beijing 100101, China; CAS Key Laboratory of Genome Sciences and Information, Beijing Institute of Genomics, Chinese Academy of Sciences and China National Center for Bioinformation, Beijing 100101, China; Human Genome Sequencing Center, Baylor College of Medicine, Houston, TX 77030, USA; National Genomics Data Center, Beijing Institute of Genomics, Chinese Academy of Sciences and China National Center for Bioinformation, Beijing 100101, China; CAS Key Laboratory of Genome Sciences and Information, Beijing Institute of Genomics, Chinese Academy of Sciences and China National Center for Bioinformation, Beijing 100101, China; National Genomics Data Center, Beijing Institute of Genomics, Chinese Academy of Sciences and China National Center for Bioinformation, Beijing 100101, China; CAS Key Laboratory of Genome Sciences and Information, Beijing Institute of Genomics, Chinese Academy of Sciences and China National Center for Bioinformation, Beijing 100101, China; National Genomics Data Center, Beijing Institute of Genomics, Chinese Academy of Sciences and China National Center for Bioinformation, Beijing 100101, China; CAS Key Laboratory of Genome Sciences and Information, Beijing Institute of Genomics, Chinese Academy of Sciences and China National Center for Bioinformation, Beijing 100101, China; National Genomics Data Center, Beijing Institute of Genomics, Chinese Academy of Sciences and China National Center for Bioinformation, Beijing 100101, China; CAS Key Laboratory of Genome Sciences and Information, Beijing Institute of Genomics, Chinese Academy of Sciences and China National Center for Bioinformation, Beijing 100101, China; National Genomics Data Center, Beijing Institute of Genomics, Chinese Academy of Sciences and China National Center for Bioinformation, Beijing 100101, China; CAS Key Laboratory of Genome Sciences and Information, Beijing Institute of Genomics, Chinese Academy of Sciences and China National Center for Bioinformation, Beijing 100101, China; National Genomics Data Center, Beijing Institute of Genomics, Chinese Academy of Sciences and China National Center for Bioinformation, Beijing 100101, China; CAS Key Laboratory of Genome Sciences and Information, Beijing Institute of Genomics, Chinese Academy of Sciences and China National Center for Bioinformation, Beijing 100101, China; National Genomics Data Center, Beijing Institute of Genomics, Chinese Academy of Sciences and China National Center for Bioinformation, Beijing 100101, China; CAS Key Laboratory of Genome Sciences and Information, Beijing Institute of Genomics, Chinese Academy of Sciences and China National Center for Bioinformation, Beijing 100101, China; University of Chinese Academy of Sciences, Beijing 100049, China; National Genomics Data Center, Beijing Institute of Genomics, Chinese Academy of Sciences and China National Center for Bioinformation, Beijing 100101, China; CAS Key Laboratory of Genome Sciences and Information, Beijing Institute of Genomics, Chinese Academy of Sciences and China National Center for Bioinformation, Beijing 100101, China; University of Chinese Academy of Sciences, Beijing 100049, China; National Genomics Data Center, Beijing Institute of Genomics, Chinese Academy of Sciences and China National Center for Bioinformation, Beijing 100101, China; CAS Key Laboratory of Genome Sciences and Information, Beijing Institute of Genomics, Chinese Academy of Sciences and China National Center for Bioinformation, Beijing 100101, China; University of Chinese Academy of Sciences, Beijing 100049, China; National Genomics Data Center, Beijing Institute of Genomics, Chinese Academy of Sciences and China National Center for Bioinformation, Beijing 100101, China; CAS Key Laboratory of Genome Sciences and Information, Beijing Institute of Genomics, Chinese Academy of Sciences and China National Center for Bioinformation, Beijing 100101, China; National Genomics Data Center, Beijing Institute of Genomics, Chinese Academy of Sciences and China National Center for Bioinformation, Beijing 100101, China; CAS Key Laboratory of Genome Sciences and Information, Beijing Institute of Genomics, Chinese Academy of Sciences and China National Center for Bioinformation, Beijing 100101, China; University of Chinese Academy of Sciences, Beijing 100049, China

**Keywords:** Nucleotide sequence, Database, GenBase, GenBank, INSDC

## Abstract

The rapid advancement of sequencing technologies poses challenges in managing the large volume and exponential growth of sequence data efficiently and on time. To address this issue, we present GenBase (https://ngdc.cncb.ac.cn/genbase), an open-access data repository that follows the International Nucleotide Sequence Database Collaboration (INSDC) data standards and structures, for efficient nucleotide sequence archiving, searching, and sharing. As a core resource within the National Genomics Data Center (NGDC) of the China National Center for Bioinformation (CNCB; https://ngdc.cncb.ac.cn), GenBase offers bilingual submission pipeline and services, as well as local submission assistance in China. GenBase also provides a unique Excel format for metadata description and feature annotation of nucleotide sequences, along with a real-time data validation system to streamline sequence submissions. As of April 23, 2024, GenBase received 68,251 nucleotide sequences and 689,574 annotated protein sequences across 414 species from 2319 submissions. Out of these, 63,614 (93%) nucleotide sequences and 620,640 (90%) annotated protein sequences have been released and are publicly accessible through GenBase’s web search system, File Transfer Protocol (FTP), and Application Programming Interface (API). Additionally, in collaboration with INSDC, GenBase has constructed an effective data exchange mechanism with GenBank and started sharing released nucleotide sequences. Furthermore, GenBase integrates all sequences from GenBank with daily updates, demonstrating its commitment to actively contributing to global sequence data management and sharing.

## Introduction

Biological research relies heavily on accurate and comprehensive DNA, RNA, and protein sequences and annotations. Therefore, specialized repositories that archive these sequences and annotations are invaluable to the worldwide research community. The International Nucleotide Sequence Database Collaboration (INSDC) [1], which includes GenBank [2] from the National Center for Biotechnology Information (NCBI) [3], the European Nucleotide Archive (ENA) from the European Bioinformatics Institute (EBI) [4], and the DNA Data Bank of Japan (DDBJ) [5] from the National Institute of Genetics, has made valuable efforts to facilitate biological research and development through sustainable maintenance and daily exchange of nucleotide data collected worldwide. Over the past decades, China has identified numerous nucleotide sequences for various species [6–9], many of which have been submitted to INSDC. Researchers in China and other countries/regions heavily rely on INSDC for sequence submission and retrieval. However, the rapid advancement of sequencing technologies has led to a significant increase in sequence data volume, posing challenges for timely and efficient submission and sharing. These challenges can slow down processes and add an increasing burden to INSDC. Additionally, time-sensitive submissions, standardized processing, and urgent release of important sequence data, as demonstrated by severe acute respiratory syndrome coronavirus 2 (SARS-CoV-2) [10,11], require database functions that offer flexibility [12,13]. Moreover, non-native English speakers may encounter language barriers during data submission, resulting in miscommunications and delays in data release. Therefore, there is an urgent need for a nucleotide data repository that adheres to INSDC rules/standards, actively exchanges data with INSDC, and provides enhanced data services for local and global researchers.

Here, we present GenBase (https://ngdc.cncb.ac.cn/genbase), an open-access data repository designed for nucleotide sequence archiving, searching, and sharing. GenBase is a core resource within the National Genomics Data Center (NGDC) [14], part of the China National Center for Bioinformation (CNCB) [15]. It adopts GenBank’s data model and supports the submission of diverse data types like messenger RNAs (mRNAs), genomic DNA, non-coding RNAs (ncRNAs), organelles, viruses, plasmids, and phages through an online bilingual submission portal that provides real-time validation for both generic and SARS-CoV-2 sequences. Additionally, GenBase integrates all sequences from GenBank with daily updates to provide free and publicly accessible data to support the distribution and sharing of the international datasets, as well as facilitate data access for Chinese researchers.

## Implementation

GenBase is implemented with Thymeleaf (a modern server-side Java template engine for both web and standalone environments; https://www.thymeleaf.org/), Spring (an application framework and inversion of control container; https://spring.io/), and MyBatis [a persistence framework with support for custom Structured Query Language (SQL), stored procedures, and advanced mapping; https://mybatis.org/mybatis-3/]. The global search function is based on the Elasticsearch module (https://www.elastic.co/elasticsearch/). Open-source data management system MySQL (https://www.mysql.com/) is used for storing metadata information.

## Database components

### Data model and accessions

**Figure 1:**
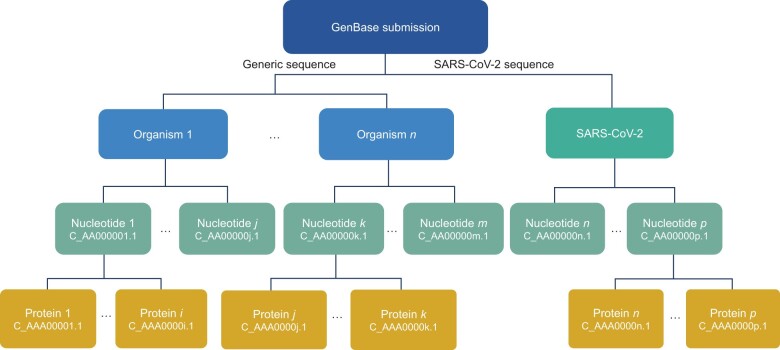
Accession assigned data model in GenBase Accession numbers are assigned in GenBase for both nucleotide and protein sequences. SARS-CoV-2, severe acute respiratory syndrome coronavirus 2.

The data model and data accessions are shown in **Figure 1**. GenBase is designed to be compatible with the INSDC data model and allows for association with two CNCB-NGDC metadata description databases [16]: BioProject (https://ngdc.cncb.ac.cn/bioproject) and BioSample (https://ngdc.cncb.ac.cn/biosample). However, it is worth noting that the linkage to these two databases is optional. GenBase allows users to submit nucleotide sequences from multiple organisms in a single batch submission. Upon submission, the system will generate a unique number with prefix “sub”. After quality control, each nucleotide sequence is assigned an accession number starting with “C_” followed by 2 letters, 6 numbers, and a sequence version number suffix. Similarly, each protein sequence associated with a given nucleotide sequence receives an accession number starting with “C_” followed by 3 letters, 5 numbers, and a sequence version number suffix. The sequence version number will be modified whenever changes are made to the sequence. Sequence records are generated and stored in the Abstract Syntax Notation dotone (ASN.1) format and displayed online in the GenBank flatfile (GBFF) format, both commonly used by GenBank [2].

### Data submission and validation

#### Generic sequences

**Figure 2:**
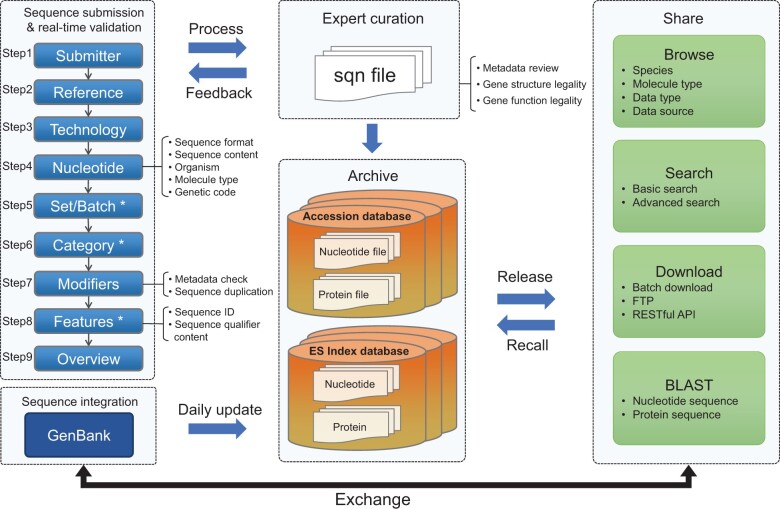
The whole architecture of GenBase The user-friendly generic sequence submission portal consists of 9 sections that enable real-time validation: Submitter, Reference, Technology, Nucleotide, Set/Batch, Category, Modifiers, Features, and Overview. An asterisk (*) indicates optional steps in SARS-CoV-2 submissions. GenBase integrates GenBank sequences for daily updates. Processed data are stored in distributed accession and ES index databases. Released sequences in GenBase are easily accessible, searchable, downloadable, and analyzable. They are also exchanged with GenBank, promoting data sharing and accessibility. ES, Elasticsearch; FTP, File Transfer Protocol; API, Application Programming Interface; BLAST, Basic Local Alignment Search Tool.

GenBase constructed a user-friendly, bilingual online portal, divided into nine sections. This interface enables comprehensive real-time validation for generic sequence submissions, including Submitter, Reference, Technology, Nucleotide, Set/Batch, Category, Modifiers, Features, and Overview (**Figure 2**).

During the “Nucleotide” phase (step 4), GenBase’s sequence validator checks the sequence files uploaded by user for sequence format, sequence content, organism, molecule type, and genetic code. Validation results are presented in a tabular format, offering detailed error information and potential correction instructions. In the “Modifiers” stage (step 7), GenBase gathers sequence metadata using an Excel-based format with 57 modifiers, affording users flexibility in metadata arrangement. A robust 18-category validation pipeline is employed to extract and validate sequence metadata. For instance, fields like Country and Organelle/Location adhere to a controlled vocabulary, while collection date and latitude follow specific formats. During the “Features” phase (step 8), GenBase optionally requests an annotation file in one of three formats: a 5-column GenBank feature table [2], Generic Feature Format (GFF), and a user-friendly Excel format developed by GenBase. Users choose one of these formats for sequence annotation, which GenBase then verifies. For example, all sequence IDs in the annotation file must match those in the nucleotide sequence file, coordinates should be integers, and gene annotations should conform to INSDC specification. Currently, 768 features with corresponding qualifiers are available for annotation [1]. After users confirm all information in the “Overview” stage, GenBase uses table2asn (https://www.ncbi.nlm.nih.gov/genbank/table2asn/) to perform a final check on each submitted sequence, generating high-quality sequence files (*e.g.*, GBFF and sqn file).

#### SARS-CoV-2 sequences

To enhance sharing efficiency for SARS-CoV-2 sequences, GenBase devised a dedicated submission module that streamlines bulk sequence submission and processing. This module follows a process similar to generic sequences, but it eliminates the need for user-submitted annotation files by using the Viral Annotation DefineR (VADR) program [17] for automatic annotation of submitted SARS-CoV-2 sequences. Additionally, GenBase provides an Excel format for SARS-CoV-2 metadata, ensuring compatibility with both INSDC and the Global Initiative on Sharing All Influenza Data (GISAID) [18]. Real-time validation is implemented throughout the entire submission process.

### GenBank sequence integration

To address the needs of the international nucleotide archive, GenBase developed an automatic pipeline for integrating nucleotide data from GenBank (Figure 2). Initially, archived GenBank data (Release 254) in aso.gz format, encompassing 1203 datasets and three description files, was fetched from the GenBank File Transfer Protocol (FTP) (https://ftp.ncbi.nlm.nih.gov/genbank/). These data were then converted into the GBK format using the asn2flat tool (https://ftp.ncbi.nlm.nih.gov/asn1-converters/by_program/asn2flat/) from the NCBI toolkit. Strict quality control scripts were utilized to extract nucleotide and corresponding protein data, yielding 261,921,660 nucleotides and 250,451,419 proteins. These files were subsequently archived into the accession database. Once all data from GenBank Release 254 was loaded, daily released aso.gz files were retrieved from the GenBank FTP and processed daily.

### Archive, release, and statistics

**Figure 3:**
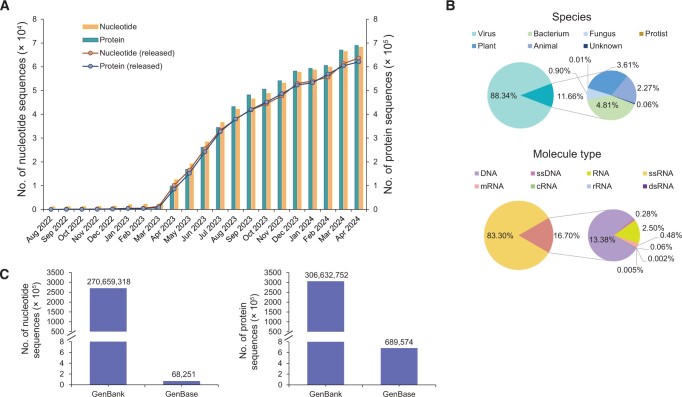
Data statistics These statistics are based on data from GenBase as of April 23, 2024. **A**. The statistics of submitted and released nucleotide and protein sequences in GenBase show a steady increase in both nucleotide and protein sequences over time. **B**. The statistics of released nucleotide sequences by species and molecule type for GenBase. **C**. The numbers of nucleotide and protein sequences directly submitted to GenBase and those integrated from GenBank. ssDNA, single-stranded DNA; ssRNA, single-stranded RNA; mRNA, messenger RNA; cRNA, coding RNA; rRNA, ribosomal RNA; dsRNA, double-stranded RNA.

GenBase maintains data integrity and enhances reusability through rule-guided automatic quality control and expert manual curation, mostly compatible with the INSDC’s data submission standards. The pipeline targets standardized data curation, assigning unique accession numbers after archiving. GenBase then generates files in GBFF and Fast All Sequences in A (FASTA) formats, storing file information in distributed accession databases and segregating metadata into Elasticsearch index databases (Figure 2). GenBase administers periodic scans of submitted sequences for ensuing publication, respecting the release dates specified by submitters. Sequences incorporated from GenBank are released daily, facilitating public browsing, retrieval, and download. Since its formal launch on March 24, 2023, GenBase has experienced a considerable influx in data submissions, leading to substantial data growth within 13 months (**Figure 3**A and B). As of April 23, 2024, GenBase incorporates and updates 270,659,318 nucleotide sequences and 306,632,752 protein sequences from GenBank (Figure 3C). It has also received 68,251 nucleotide sequences and 689,574 annotated protein sequences from 271 submitters covering 414 species (Figure 3C). Of the submitted data, 63,614 nucleotide sequences (93%) and 620,640 annotated protein sequences (90%) have been made available to the public. Notably, out of 55,514 submitted SARS-CoV-2 genome sequences with standardized annotations, 52,758 have been released. In collaboration with INSDC, GenBase has started sharing released nucleotide sequences with GenBank (*e.g.*, https://www.ncbi.nlm.nih.gov/nuccore/OQ716536 for C_AA001251 and https://www.ncbi.nlm.nih.gov/nuccore/OR036766 for C_AA014956), underscoring its commitment to the global sequence data exchange.

### Data retrieval and download

In GenBase, users can utilize either a basic or advanced search function with 31 search conditions (*e.g.*, accession, organism, gene name, and journal). The advanced search maintains a history for easy tracking of data retrieval. Users can refine search results using filters such as species, data source, and data types. Sorting options (*e.g.*, accession, modified date, organism, and sequence length) help organize results efficiently. GenBase provides four data display formats (summary, GBFF, FASTA, and accession list) and supports batch downloading to meet diverse usage requirements. To facilitate streamlined batch download of FASTA files, a RESTful Application Programming Interface (API) (such as https://ngdc.cncb.ac.cn/genbase/api/file/fasta?acc=C_AA004835.1) has been developed. Additionally, an FTP site (https://download2.cncb.ac.cn/genbase/daily/) is accessible for daily releases of nucleotide and protein sequences.

## Summary and future directions

GenBase is a comprehensive platform that offers a range of impressive functions for archiving, sharing, and searching nucleotide sequences. Firstly, it supports both individual and bulk sequence submissions, assigning accessions and tracking version changes for each submission. This enables sequence publishing and archiving, and facilitates data sharing among users. Secondly, GenBase provides multiple access options for browsing, retrieving, and downloading data, meeting the conventional requirements for sequence applications. Users can easily access the data that they need in various ways. Thirdly, GenBase incorporates sequence analysis tools such as Basic Local Alignment Search Tool (BLAST) [19], making it convenient for users to analyze sequences within the platform. In addition, GenBase establishes linkages with several databases in NGDC, including the metadata databases BioProject and BioSample, as well as the literature database OpenLB (https://ngdc.cncb.ac.cn/openlb/home). This feature allows for user-friendly data tracking and enhances the overall user experience. Furthermore, GenBase offers bilingual submission pipeline and services, as well as local submission assistance in China. These services improve communication efficiency and make the platform more accessible to users. Lastly, GenBase establishes a good platform to integrate sequences from INSDC with daily updates, thus making it very convenient for users in China and other countries to access these data — a practice encouraged and welcomed by INSDC.

Although the design and functions of the database are similar to those of INSDC’s DDBJ, ENA, and GenBank, GenBase has its significance and unique features. The unique implementation of the Excel format for metadata description and feature annotation of nucleotide sequences, along with our real-time data validation system, simplifies and streamlines the sequence submission process within GenBase. Meanwhile, the availability of bilingual submission pipeline, local services, and the short turnaround time for data exchange with GenBank, have attracted a large number of sequence submissions to GenBase. As a result, many sequences, including important ones such as those for SARS-CoV-2, become publicly available to the broad research community. The exponential growth in nucleotide sequence submissions highlights the significant potential of GenBase as an important resource for advancing worldwide biological research.

In the future, GenBase is committed to advancing biological research and development through ongoing efforts, including improving web interfaces for data submission, retrieval, and presentation. Our future plans include expanding services to encompass genome annotation, with a specific emphasis on virus, mitochondrial, and chloroplast genomes, ensuring accuracy in downstream data analysis. Additionally, we aim to integrate user-friendly online tools for convenient sequence data analysis, such as species identification. Finally, we strive to promote collaboration by sharing and exchanging all publicly available nucleotide sequences with INSDC members, thereby providing comprehensive data resources for researchers worldwide.

## Data Availability

GenBase is freely accessible at https://ngdc.cncb.ac.cn/genbase.

## CRediT author statement

**Congfan Bu:** Investigation, Methodology, Writing – original draft. **Xinchang Zheng:** Investigation, Methodology, Writing – original draft. **Xuetong Zhao:** Investigation, Data curation, Writing – original draft, Writing – review & editing. **Tianyi Xu:** Investigation, Writing – original draft. **Xue Bai:** Data curation, Writing – original draft. **Yaokai Jia:** Investigation, Software. **Meili Chen:** Investigation, Writing – original draft. **Lili Hao:** Project administration, Investigation. **Jingfa Xiao:** Conceptualization, Writing – review & editing. **Zhang Zhang:** Conceptualization, Writing – review & editing. **Wenming Zhao:** Conceptualization, Writing – review & editing. **Bixia Tang:** Investigation, Methodology, Software, Writing – original draft, Writing – review & editing. **Yiming Bao:** Conceptualization, Writing – review & editing, Supervision. All authors have read and approved the final manuscript.
